# Masking Ability of Bleach Shade Composite Resins in Different Thicknesses

**DOI:** 10.1155/2022/7479299

**Published:** 2022-05-17

**Authors:** Haleh Valizadeh-Haghi, Shadieh Molaee, Aziz Kamran, Shiva Davoodzadeh

**Affiliations:** ^1^Department of Operative Dentistry, Ardabil University of Medical Sciences, Ardabil, Iran; ^2^School of Medicine and Allied Medical Sciences, Ardabil University of Medical Sciences, Ardabil, Iran; ^3^School of Dentistry, Ardabil University of Medical Sciences, Ardabil, Iran

## Abstract

**Introduction:**

In recent years, the use of bleach shade composite resins has increased due to the high esthetic demands. The aim of the present study was to investigate the masking ability and translucency of several bleach shade composite resins.

**Materials and Methods:**

In this in vitro study, 180 discs from 9 bleach shade composite resins of 5 brands (G-aenial Anterior, Gradia Direct, Charisma Diamond, Estelite Sigma Quick, and Renamel Microfill) in thicknesses of 0.5, 1, 1.5, and 2 mm were prepared. The color parameters of the specimens were measured in a CIE l^*∗*^a^*∗*^b^*∗*^ system using a spectrophotometer. Masking ability and translucency parameters were calculated, and the influence of thickness, shade, and composite resin brand on these variables was analyzed using the Kruskal–Wallis and Mann–Whitney *U* tests.

**Results:**

Both masking ability and translucency differed significantly among groups (*p* < 0.001), but only MA was different between different brands (*p* < 0.001). Higher thicknesses showed lower translucency in all pairwise comparisons (*p* < 0.001). 0.5 mm-thick specimens had lower masking ability than 1.5 mm- (*p*=0.016) and 2 mm- (*p*=0.035) thick ones. However, masking ability of other thickness pairs did not show a significant difference (*p* > 0.05). G-aenial Anterior and Renamel Microfill had the highest and lowest masking ability. Bleach white shades had lower masking ability and higher translucency than extrawhite bleach shades.

**Conclusion:**

Translucency and masking ability of bleach shade composite resins differed in different thicknesses, composite resin brands, and shade groups.

## 1. Introduction

Nowadays, composite resins are widely used as restorative materials in smile frames and provide satisfying esthetic results for both dentists and patients. On the other hand, they have economic advantages compared to conventional prosthetic treatments [1–3]. For the success of composite restorations, it is esthetically necessary that the restoration has a color similar to the adjacent dental structure and is in harmony with it [4]. Color matching in composite resin restorations is not an easy task, especially in cases of severe tooth discoloration and in through and through restorations, as in these situations there is not enough or normal adjacent tooth structure and usually a grayish appearance will be seen in the final composite restoration, which fails color matching [5]. Complete masking of tooth discoloration through direct restorations is a complex procedure due to the translucency of the composite resins [6–8] and requires careful diagnosis, treatment planning, and the use of the correct restorative technique to reach the acceptable outcomes [9].

Translucency is defined as the ability of a substance to pass through the light and is in between a range from complete opacity to transparency [10]. Another property which is inversely related to translucency is masking ability. It is the ability of a substance to mask the background discoloration [11–13]. Studies have shown that both two properties are affected by the thickness of the material [14, 15]. It is obvious that in clinical situations, it is almost impossible to create a uniform thickness of the esthetic restoration, so according to the condition of the restored tooth like probable discoloration and the optical properties of the selected restorative material, the esthetic appearance can be affected [16]. Studies showed that the optical characteristics of different shade groups of composite resins produced by different manufacturers are material-specific [17–20], and as a result, the study of common composite resin brands available in a country's market can be valuable [21]. It was reported that the translucency and color of a restorative material are determined not only by macroscopic features such as filler [22] and matrix composition [22, 23] and the size [24] and content of dispersed fillers [23, 25, 26] but also by the amount of pigments and other chemical ingredients of the material [18].

On the other hand, the use of bleach shade composite resins has highly increased in the recent years as the patients demand high esthetic levels and the treatment modalities like tooth whitening are more available these days [27, 28]. In addition, it has been reported that the shade of composite resin can affect the optical properties [29–31]. However, few studies have been conducted on the optical properties of bleach shade composites [32]. In a recent study, which examined the masking ability of bleach shade composite resins in different layering techniques, it was observed that they cannot mask the different background colors [33].

Due to the lack of studies about bleach shade composite resins, the aim of the present study was to compare the masking ability and translucency of some commercially available bleach shade composite resins, which are common in Iran's dental market and to investigate the effect of thickness, shade, and brand of composite resin on the translucency and masking ability of the materials. The hypotheses were as follows:

1. Translucency and masking ability differ in groups with different shades, brands, and thicknesses
2. Translucency and masking ability of composite resins of similar shades are different in different brands
3. Higher thicknesses correlate with lower translucency and higher masking ability
4. Lighter shades correlate with lower translucency and higher masking ability

## 2. Materials and Methods

This in vitro study was performed on the composite resin discs (*n* = 180). Composite resin materials used in the fabrication of discs were as follows: one microfill composite resin (Renamel Microfill (Cosmedent Inc, USA)), two microhybrid composite resins (Gradia Direct (GC, Japan) and G-aenial Anterior (GC, Japan)), and two nanohybrid composite resins (Estelite Sigma Quick (Tokuyama Dental, Japan) and Charisma Diamond (Heraeus Kulzer, Germany)). Two universal bleach shades (BW or BL or SB3 and XBW or BXL or SB2) of these composite resins were selected. Discs were made in 4 different thicknesses (0.5, 1, 1.5, and 2 mm) in each shade and composite group (*n* = 5).  Table 1 summarizes the short name of composite groups used in the study. The sample size of 5 chosen brands was based on a previous similar study [14], and GPower software was used at a power of 0.95 and *α* = 0.05.

### 2.1. Specimen Preparation

To prepare the specimens, cylindrical Teflon molds with the height of 0.5, 1, 1.5, and 2 mm and an inner diameter of 5 mm were placed on a polyester strip on a glass slab. The composite resin material was filled in the mold, covered with a second polyester strip and a second glass slab, and light-cured on both sides for 40 sec using an LED-curing unit (cordless LED curing light B (Woodpecker, China)) with an intensity of 1000 mW/cm^2^. Then, the specimens were polished using 1000, 1200, and 2000 grit silicon carbide paper. To complete the polymerization, the specimens were incubated in distilled water at 37°C for 24 h.

### 2.2. Color Measurement and Calculating Masking Ability and Translucency

The color measurement was performed by using a YS3020 diffuse8 (3nh, China) spectrophotometer with a D65 standard light source. A black opaque box was used to remove ambient light. The CIE L^*∗*^a^*∗*^b^*∗*^ color coordinates of each specimen placed on a black and white background were measured, and an average of three measurements was recorded for each specimen.

The color change formula used in different parts of the study was the CIEDE2000 color difference (∆E00) formula as follows: ∆E00 = [(∆L'/KLSL)^2^ + (∆C'/KCSC)^2^ + (∆H '/KHSH)^2^ + RT (∆C'/KCSC) (∆H'/KHSH)]^1/2^, where ∆L', ∆C, and ∆H′ were the amount of lightness, chroma, and hue differences. SL, SC, and SH were the weighting functions, and RT was the function of interaction between differences in chroma and hue in the blue region. KL, KC, and KH were the parametric factors set at 1 [34].

Masking ability (MA) was obtained by measuring the values of L^*∗*^, a^*∗*^, and b^*∗*^ of the specimens placed on a black background and calculating the difference with the intrinsic color of the same composite resin using the color change formula. To obtain the intrinsic color of the composite resin, 3 specimens of each composite resin with a thickness of 4 mm were prepared and the L^*∗*^, a^*∗*^, and b^*∗*^ values of the specimens were measured on a gray background. Then, the average value was considered as the intrinsic color. According to the color change formula, the smaller numeric value means that the color of the specimen is closer to its intrinsic color and the specimen could mask the black background, so the smaller the numeric value, the higher the MA. CIEDE2000 50 : 50% perceptibility (∆E00 = 0.8) and acceptability (∆E00 = 1.8) thresholds were considered in this study [35].

In addition to masking ability, the translucency parameter (TP) of specimens was also calculated. For this purpose, values of L^*∗*^, a^*∗*^, and b^*∗*^ of the specimens were measured on both black and white backgrounds and the color difference between the two measurements was considered as TP.

The backgrounds used in this study include a black background (b: 1.23, a: 0.47, and L: 30.34), a white background (b: −7.94, a: 1.05, and L: 92.50), and a gray background (b: −3.44, a: −2.22, and L: 41.34).

Table 2 shows the composition of the composite resins used in the study.

### 2.3. Statistical Analysis

Data were analyzed with SPSS 23 statistical software (IMB, USA). Descriptive values of MA and TP in 4 thicknesses were reported in different composite resin groups. Distribution of data was checked using the Kolmogorov–Smirnov test. The Kruskal–Wallis and Mann–Whitney *U* tests were used to compare MA and TP between different groups. Type 1 statistical error was set at 0.05.

## 3. Results

**Figure 1:**
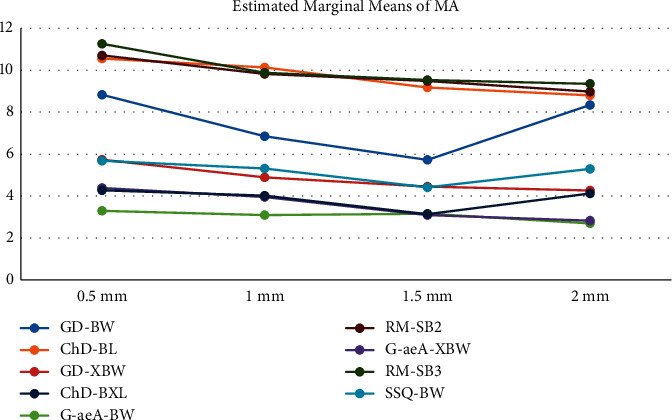
MA of different groups.

**Figure 2:**
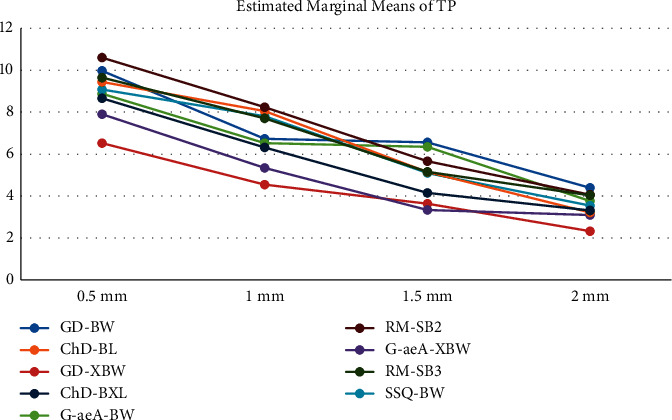
TP of different groups.

Figures 1 and 2 show the mean values of MA and TP in different thicknesses in the studied composite resins. The maximum and minimum MA were observed in 2 mm G-aeA-BW (2.7 ± 0.6) and 0.5 mm RM-SB3 (11.3 ± 1.0), respectively. The numeric amount of MA in all groups was higher than 50 : 50% perceptibility and acceptability levels; in other words, none of the specimens were able to mask the black background. In the case of TP, the lowest value of 2.3 ± 1.0 was obtained in 2 mm GD-XBW and the highest value of 10.6 ± 1.0 was obtained in 0.5 mm RM-SB2.

Rejecting the normal distribution of data in different groups by the Kolmogorov–Smirnov test, the nonparametric tests were used. To evaluate the first hypothesis of the study, the Kruskal–Wallis test was used and showed significant differences in both MA and TP between different groups (*p* < 0.001).

To analyze the effect of composite brands, the values of MA and TP were compared between different brands using the Kruskal–Wallis test. The test showed that different brands had different MA (*p* < 0.001) but not significant different TP (*p*=0.15). Pairwise comparisons regarding MA of different brands were performed using the Mann–Whitney *U* test. The following trend was observed: MA of G-aeA > GD = ESQ = ChD > RM.

MA and TP in different thicknesses were compared using the Kruskal–Wallis test and showed a significant difference in TP values (*p* < 0.001); however, a borderline significant difference in MA values was observed (*p*=0.057). Pairwise comparisons of TP in different thicknesses using the Mann–Whitney *U* test showed that TP was less in higher thicknesses in each pair (*p* < 0.001). About the MA, although it increased generally with the increase in thickness, in most pairwise comparisons (Table 3), there were not statistically significant differences (*p* > 0.05).

According to the results of the Mann–Whitney *U* test, the MA of BW, BL, and Sb3 shades was less than that of XBW, BXL, and Sb2 shades (*p* < 0.001). Also, the TP values of BW, BL, and Sb3 shades were higher than those of XBW, BXL, and Sb2 shades (*p*=0.004).

## 4. Discussion

In the present study, the masking ability and translucency of several bleach shade composite resins of different thicknesses were investigated and the results showed that MA and TP differed in various groups, so, the first hypothesis was accepted as shown in different studies [4, 5, 12, 14, 17–21]. The CIEDE2000 color change formula used in this study utilizes important adjustments of color coordinates in order to approximate how color change is perceived by the human eye and is a more recent and appropriate formula compared to the CIE L^*∗*^a^*∗*^b^*∗*^ color change formula in dentistry [31]. However, still the latter is frequently used in studies [14]. The results of a recent study showed the same trend for both formulas but with different absolute values [31].

Regarding the effect of the brand of composite resins on TP and MA, results showed different trends in MA and TP. Although there was not a significant difference between TP values of different brands, the MA was higher than others in G-aeA and lower in RM brand. Different results were obtained in studies, and several authors have shown that the optical properties depend on the manufacturers [17–20]. The type of filler and the difference between the refractive index of the filler and resin are other influencing factors in masking ability and translucency of a composite resin [22]. Also, the amount of filler and the ratio of matrix and filler are effective in optical properties [23]. As G-aeA is a microhybrid and RM is a microfill composite resin, the results of the present study can be explained by the size and amount of the filler. However, the results contradict a previous study which reported that microhybrid composite resins have more translucency than microfill ones [24]. In another study, the masking ability of nanohybrid composite resins was higher than that of microhybrid ones [14], which contradicts the results of the present study. Furthermore, researchers showed that if the filler size is constant, the higher filler load will lead to the lower translucency [25]. However, in another study, no significant relationship was observed between the filler load and translucency [20]. It seems that all factors include the volume concentration, type, and size of the filler as long as the type of resin monomer, which influences the refractive index, can affect the masking ability and translucency [26]. Considering that the observed pattern on the masking ability of G-aeA and RM was not observed in other microhybrid and nanohybrid composite resins in the present study as they show similar MA, it can be concluded that other ingredients in these composite resins and different combinations of resin and filler types can play a more important role than the filler size classification. As shown in studies, the masking ability in composite resins is achieved by adding some ingredients, typically inorganic oxides. However, the pigment content is not revealed by manufacturers and usually remains secret. In addition, it is expected that less-translucent composite resins have increased inorganic filler content, while it should be noticed that manufacturers often report a general range of the filler content for their composite systems and do not provide small differences between the different shades of one system [18]. Also, the pigments and variety of additives in the composite resins of different manufacturers have significant effects [19].

The results of the present study showed that in the higher thicknesses of a composite resin, lower TP values were observed, but in the case of MA, no significant differences were found in some thicknesses, although there was still a general tendency to increase masking ability by increasing the thickness. This result in the studied bleach shade composites is in agreement with previous studies on the nonbleach shade ones, as they have shown that by increasing the thickness of a composite resin, masking ability increases [12, 15, 21] and translucency decreases [12, 14].

About the last hypothesis of the study regarding the effect of the specific shade, results showed that the lighter colors (XBW and Sb2) have lower translucency and higher masking ability, which confirmed the hypothesis. It can be attributed to the presence of more opacifiers for more lightening effects. In various studies, although performed on the nonbleach shade composite resins, TP and MA have been dependent on the shade of composite resin systems [12, 29, 30], but different results were obtained. In a study, lighter shades had lower translucency [12] similar to the present study. However, another study showed that A1 shades of composite resins of each brand had higher translucency than darker shades toward A3.5 [29]. It seems that different pigments and opacifiers added to reach the desired shades of composite resin systems play a role in different outcomes [19].

Considering the clinically acceptable threshold, none of the studied bleach shade composite resins up to 2 mm thickness could mask the black background, which is in accordance with a previous study [32]. However, it seems that the optical properties of composite resin materials depend on a set of different factors and sufficient knowledge of clinicians about the optical properties of various composite resins along with his/her own experience is crucial in selecting the appropriate material and offering a successful restoration [4].

Slightly different trends in TP and MA of different composite brands and thicknesses were found in this study that can be attributed to the sample size or to the different calculation method of each parameter. Intrinsic color in the present study, which is used in the MA formula, was obtained by calculating the mean color values measured in 3 prepared discs of each composite resin group and was entered as a fixed number in the MA formula. It can be expected that this manner of measurement of intrinsic color which was not performed in the same specimen which underwent the MA measurement could have a small effect on the results. On the other hand, the different results between MA and TP were observed in other studies as well, including a study in which a significant difference was found between the masking ability of Charisma A2 composite resin and Estelite Sigma Quick OA3, while there was not any significant difference between their translucency [12]. TP is a mathematical calculation defined as the color difference of a material on the black and white backgrounds, while MA is the actual masking ability of the material and seems to be a better and more useful indicator than TP in clinical judgments as it directly measures the color change between the intrinsic color of a composite resin and the color of the specimen placed on a colored background [4, 12, 13]. It has been said that TP alone cannot completely determine the ability of a specimen to mask the background color [4]. Furthermore, performing other studies on the ability of bleach shade composite resin in different backgrounds such as discolored teeth is highly recommended.

One of the strengths of the current study was the use of CIEDE2000 color change formula in order for the results to be more close to the human eye perception. In addition, the study of 36 groups of different shade, brand, and thickness combinations of commercially available bleach shade composites which cover a wide range of commonly used composites in the practice was another good point. However, the study had some limitations, including the use of only one discolored background (black background), performing color measurements only in one timeline (24 h after polymerization), and not considering the effect of aging on the optical properties. Continuing the study on the masking ability of these groups of composites against different discolored substrates is highly recommended.

## 5. Conclusion

Within the limitations of this study, the following was concluded:

1. The translucency and masking ability in bleach shade composite resins differed in different groups of shades, brands, and thicknesses
2. Different brands studied here had different masking abilities but the same translucency values
3. G-aenial Anterior had the highest masking ability, and Renamel Microfill had the lowest one among others
4. Thicker specimens had lower translucency, but a constant manner of the increase was not obtained about masking ability
5. Extrawhite shades had higher masking ability and lower translucency values than white shades

## Figures and Tables

**Table 1:** Short names of composite groups.

Composite brands	Shade	Short names of composite groups
Renamel Microfill	Superbright2	RM-SB2
	Superbright3	RM-SB3

Gradia Direct	Bleach white	GD-BW
	Extra bleach white	GD-XBW

G-aenial Anterior	Bleach white	G-aeA-BW
	Extra bleach white	G-aeA-XBW

Estelite Sigma Quick	Bleach white	ESQ-BW

Charisma Diamond	Bleach light	ChD-BL
	Extra bleach light	ChD-BXL

**Table 2:** Materials used in the study.

Composite	Manufacturer	Composition
Gradia Direct	GC, Japan	Microhybrid composite resin matrix: urethane dimethacrylate (UDMA), dimethacrylates, trimethacrylates Fillers: silica and prepolymerised resin fillers (73 wt%)

G-aenial Anterior	GC, Japan	Microhybrid composite matrix: UDMA, dimethacrylate comonomers Fillers: prepolymerised silica and strontium fluoride containing fillers (76 wt%)

Estelite Sigma Quick	Tokuyama Dental, Japan	Nanohybrid composite matrix: Bis-GMA, TEGDMA fillers: silica-zirconia fillers, silica-titania fillers (82 wt%)

Charisma Diamond	Kulzer, Germany	Nanohybrid composite matrix: TCD-DI-HEA, UDMA, Bis-GMA, TEGDMA Fillers: Ba, Al, F glass, and colloidal silica (77 wt%)

Renamel Microfill	Cosmodent, USA	Microfill composite matrix: diurethane dimethacrylate, butanediol dimethacrylate, multifunctional methacrylate ester Fillers: pyrogenic silicic acid filler (60 wt%)

**Table 3:** The results of the Mann–Whitney *U* test: comparisons of MA of different thicknesses.

Thickness 1 (mm)	Thickness 2 (mm)	Mean rank of thickness 1	Mean rank of thickness 2	*p* value
0.5	1	49.3	41.7	0.166
	1.5	52.1	38.9	0.016
	2	51.3	39.7	0.035

1	1.5	48.8	42.2	0.225
	2	48.7	42.3	0.250

1.5	2	44.9	46.1	0.824

## Data Availability

The data are available from the corresponding author upon request via email (hvh_haleh@yahoo.com).

## Additional Points

Color matching of composite restorations is a task that needs comprehensive knowledge about the optical properties of different materials, of which are masking ability and translucency that influence the masking of underlying tooth or darkness of the oral cavity by restoration. Nowadays, bleach shade composites are used more frequently, so the study of optical properties of these materials can be helpful.
